# Systematic detection of tertiary structural modules in large RNAs and RNP interfaces by Tb-seq

**DOI:** 10.1038/s41467-023-38623-1

**Published:** 2023-06-09

**Authors:** Shivali Patel, Alec N. Sexton, Madison S. Strine, Craig B. Wilen, Matthew D. Simon, Anna Marie Pyle

**Affiliations:** 1 Department of Molecular Biophysics and Biochemistry, Yale University, New Haven, CT USA; 2 Department of Laboratory Medicine, Yale School of Medicine, New Haven, CT USA; 3 Department of Immunobiology, Yale School of Medicine, New Haven, CT USA; 4 Chemical Biology Institute, Yale University, West Haven, CT USA; 5 Howard Hughes Medical Institute, Chevy Chase, MD USA; 6 Department of Chemistry, Yale University, New Haven, CT USA; 7 Department of Molecular, Cellular, and Developmental Biology, Yale University, New Haven, CT USA

**Keywords:** RNA, Chemical tools, RNA sequencing, Structural biology, High-throughput screening

## Abstract

Compact RNA structural motifs control many aspects of gene expression, but we lack methods for finding these structures in the vast expanse of multi-kilobase RNAs. To adopt specific 3-D shapes, many RNA modules must compress their RNA backbones together, bringing negatively charged phosphates into close proximity. This is often accomplished by recruiting multivalent cations (usually Mg^2+^), which stabilize these sites and neutralize regions of local negative charge. Coordinated lanthanide ions, such as terbium (III) (Tb^3+^), can also be recruited to these sites, where they induce efficient RNA cleavage, thereby revealing compact RNA 3-D modules. Until now, Tb^3+^ cleavage sites were monitored via low-throughput biochemical methods only applicable to small RNAs. Here we present Tb-seq, a high-throughput sequencing method for detecting compact tertiary structures in large RNAs. Tb-seq detects sharp backbone turns found in RNA tertiary structures and RNP interfaces, providing a way to scan transcriptomes for stable structural modules and potential riboregulatory motifs.

## Abstract (web summary)

Compact RNA structural motifs control many aspects of gene expression, but methods for their identification are lacking. Here the authors present a sequencing-based terbium probing approach to detect complex 3D structural elements, which can be used to pinpoint potential riboregulatory elements.

## Introduction

RNAs can adopt complex folded motifs and higher-order 3-D structures that are essential across a variety of specific cellular processes^1–5^. It has recently become clear that many types of multi-kilobase RNA transcripts contain regions of tertiary structure that, either alone or in concert with protein partners, carry out biological function^6,7^. However, identifying these regions of complex RNA structure remains challenging. Current structure prediction methods on long RNAs are unable to pinpoint regions containing stable RNA tertiary structure modules or complex protein binding sites from sequence alone^8^. While biophysical techniques such as NMR^9^, x-ray crystallography^10^ and cryo-EM^11^ are invaluable tools for the observation of RNA structure, they are time-consuming and difficult to perform on a multikilobase-length RNA that contains a mixture of both structured and flexible regions. As our understanding of their biological functions becomes increasingly important, and interest in small molecule targeting of RNAs grows, it is vital to develop tools for identifying regions of tertiary structure in long RNA molecules.

In recent years, chemical probing has become a powerful tool for studying RNA structure. Many important advances have improved our ability to identify single- versus double-stranded nucleotides in RNA^12–16^, and these data have primarily been used to infer secondary but not tertiary structures of RNA. Fewer methods have been developed to detect higher order structure and these protocols are limited to an assessment of solvent accessible regions^17–19^ or identification of long-range RNA-RNA base-pairs by cross-linking methods^20,21^ or statistical correlations in mutational profiling^22,23^. The field would benefit from a readily adaptable, high throughput approach for identifying regions of local tertiary structure, which are often hallmarks of functional RNA motifs and riboregulatory elements.

**Fig. 1:**
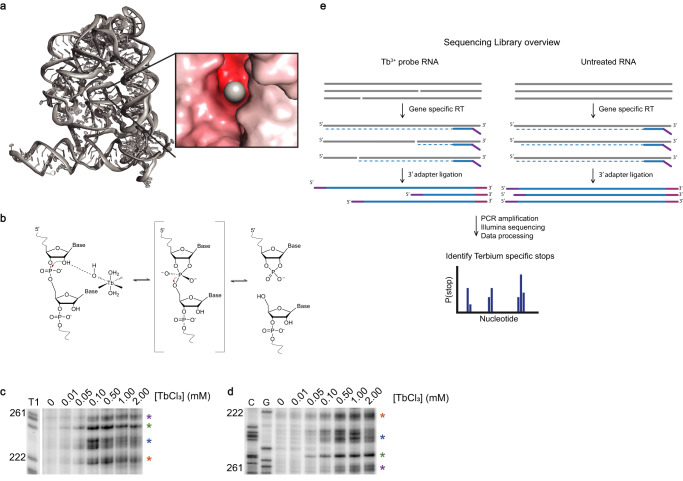
Developing a sequencing-based approach to detect Tb^3+^ cleavage sites. **a** 3D structure of group II intron with insert showing localization of metal ion in region of negative electrostatic potential region Adapted from PDB “4E8M”. **b** Mechanism of Tb^3+^ mediated cleavage. **c** Denaturing electrophoresis of ^32^P-labeled aI5γ RNA probed at the indicated TbCl_3_ concentrations. Source data are provided as a Source Data file. *N* = 2. **d** Primer extension electrophoresis of corresponding cDNA products from reverse transcription. Source data are provided as a Source Data file. *N* = 2. **e** Tb-seq library preparation workflow. A Tb^3+^ cleaved RNA and an untreated RNA are reverse transcribed with MarathonRT and a gene-specific RT primer containing a 5’ adapter handle. Illumina adapter (purple). The resulting cDNAs are ligated to a random hexamer fused to an Illumina adapter (pink) and PCR amplified to incorporate Illumina multiplex handles. Stop sites are processed using the RTEvents counter script (see Methods).

High-resolution RNA structures show that regions of tightly packed tertiary structure often contain phosphate backbones that are packed in close proximity, within the same strand or on adjacent strands. These local regions of intense negative electrostatic potential act as sinks for multivalent ion coordination^24,25^ (Fig. 1a). One way to probe these electrostatically negative reservoirs is to monitor the cleavage patterns catalyzed by coordinated metal ions^26^. When nucleotides in such regions adopt an “in-line geometry”, which aligns an upstream 2’-OH with the downstream 3’-OR group of a phosphodiester linkage^27^, adjacent metal hydroxide ions can behave as a general base, deprotonating the 2’-OH group and producing a 2’ oxyanion nucleophile that attacks the adjacent phosphate and causes strand scission^27^. While this type of Mg^2+^-catalyzed cleavage (known as in-line probing^28^) normally occurs on a slow timescale that ranges from hours to days^28^, the same phenomenon is greatly accelerated by lanthanide ions such as terbium (Tb^3+^) and europium (Eu^3+^)^26,29^. Tb^3+^ and Mg^2+^ share similar ionic radii (0.92 Å and 0.72 Å, respectively)^26^ and coordination geometry preferences for oxygen^30^, but lanthanide ions have an additional positive charge and the pKa of coordinated water molecules is much lower for ions such as Tb^3+^, (pKa ~7.9 for Tb^3+^-H_2_O versus ~11.4 for Mg^2+^-H_2_O)^30,31^. Therefore, at low ion concentrations and neutral pH, Tb^3+^ coordinates with structured RNA binding sites in a manner that is similar to that of Mg^2+^ ^32–35^_,_ but the more potent Tb^3+^ general base rapidly facilitates RNA backbone cleavage at sites of metal ion binding^31^ (Fig. 1b). Tb^3+^ probing of RNA has been used extensively in the past, but until now, it was a low-throughput method that relied on electrophoretic quantification^30,32,36^.

Here we present Tb-seq, a sequencing-based approach that employs Tb^3+^ to detect regions of tertiary structure in long RNAs. To demonstrate the efficacy of this technique, we first apply the Tb-seq pipeline to identify tertiary structural motifs within structurally well-characterized RNA molecules. We then apply it to probe known and unknown RNA structures in a cellular context to investigate RNA motifs and protein binding sites. These studies show that Tb-seq detects regions of RNA involved in RNA tertiary structure motifs and within RNP complexes, thereby providing a powerful approach for pinpointing regions of complex RNA structure that are potentially associated with RNA functional elements.

## Results

### Developing a high throughput sequencing-based approach to detect Tb^3+^ cleavage sites

To precisely identify tertiary RNA structural elements in a high throughput manner, we adapted a previously established Tb^3+^ RNA cleavage assay for accurate single nucleotide detection in an RNA of interest. In the classical version of this experiment, the RNA of interest is end-labeled with ^32^P, probed with Tb^3+^ and the sites of hydrolysis are visualized after electrophoresis of the RNA^30,32,33,36^. To adapt this assay to a sequencing readout, we first determined if the expected Tb^3+^cleavage sites could be detected as termination events upon reverse transcription (RT) with a processive reverse transcriptase, MarathonRT^37^. We used the D135 ribozyme derived from yeast group II intron aI5γ, which has been extensively characterized using the classical version of Tb^3+^ probing^36^. We found that reverse transcription stops (Fig. 1c) recapitulate the previously-published Tb^3+^ cleavage pattern (Fig. 1d), thereby validating RT as a tool to detect Tb^3+^-induced cleavage. We then adapted this approach for NGS sequencing. Specifically, a Tb^3+^ cleaved RNA or an untreated RNA is reverse transcribed with a gene-specific RT primer containing a 5’ adapter handle. The resulting cDNA is 3’ adapter-ligated and PCR amplified to add Illumina multiplex handles. We then implemented a previously-developed pipeline for assessing RT termination events^38^ to quantify termination (Fig. 1e). This sequencing and analysis approach, Tb-seq, recapitulated the previously published D135 Tb^3+^ cut sites. (Supplementary Fig. 1). To better understand whether Tb-seq could be used as a discovery tool for assessing higher-order RNA structure in a variety of RNA types, we next applied this method to evaluate the patterns of cleavage in RNAs with well-determined tertiary structures.

### Tb-seq reveals well-folded RNA tertiary elements

To benchmark Tb-seq on RNAs that have never been analyzed with Tb^3+^ cleavage before, we probed in vitro transcribed RNAs that contain both well-folded RNA tertiary elements and known metal sites. We chose a group II intron from *Oceanobacillus iheyensis* (*O.i*.) that has been well characterized biochemically and crystallographically^39^. First, reagent concentrations and reaction times were optimized to obtain an ideal reactivity signal and ensure the RNA is not over-cleaved (Supplementary Fig. 2). We performed Tb-seq using a range of Tb^3+^ concentrations from 0.01 mM - 2 mM for 10 min in order to evaluate the intensity and location of cleavage patterns (Supplementary Fig. 3). We observe that the cleavage signal is abolished if the *O.i*. intron is denatured prior to probing, supporting our interpretation that cleavage signals are indicators of RNA structure. To determine whether secondary structure alone is sufficient to produce the cleavage pattern, the intron was folded only in the presence of monovalent ions, under conditions lacking the magnesium ions known to promote its characteristic tertiary structure^40^ (Supplementary Fig. 4A). We found that secondary structure was insufficient to establish the signals, supporting our interpretation that Tb-seq signals correspond to sites of tertiary structure. Instead, we found that at certain Tb^3+^ concentrations (0.5 mM), non-specific cleavage is observed (Supplementary Fig. 4B). These results demonstrate that a correctly folded intron-containing well-defined tertiary elements is required for Tb^3+^ coordination and site-specific RNA cleavage.

**Fig. 2:**
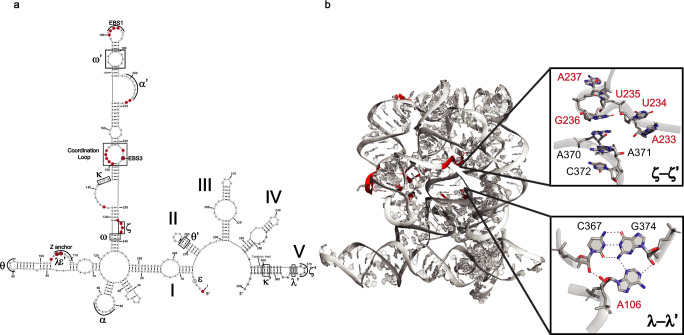
Tb-seq of *O.i*. intron detects long-range, evolutionarily conserved RNA-RNA interactions. **a** Secondary structure of the *O.i*. intron displaying sites of strong Tb^3+^cleavage (red). Long-range RNA-RNA interactions are indicated by Greek letters. EBS1 and EBS2 correspond to exon binding sites. Gray nucleotides indicate a lack of sequencing data in this region. **b** 3-D structure of *O.i*. intron showing sites of strong Tb^3+^ cleavage on the RNA backbone (red highlight). Inserts showing close-up view of two long-range RNA-RNA interactions (ζ-ζ’ and λ-λ’) with nucleotides that display Tb^3+^ cleavage (red) and hydrogen bonds (purple dashed lines). Adapted from PDB “4E8M”. Source data are provided as a Source Data file.

Next, we established a three-point criteria set for selecting nucleotide stop sites that are likely to result from specific, site-bound Tb^3+^-dependent cleavage, which we will call “strong Tb signal”. First, a reactivity value of >0.5 was established for detecting strong sites of cleavage and maximizing probe specificity (see Methods). Second, these sites must be observed in two independent replicates to demonstrate reproducibility. Third, selected sites must show a dependence of signal on Tb^3+^ concentration to ensure that stop signals are not due to spontaneous RT termination events. Nucleotide sites that satisfy these criteria are highlighted in red in the secondary structure diagram of the *O.i*. intron (Fig. 2b). Upon initial inspection, we observed that the strongest Tb^3+^ sites are in short-loop regions within the RNA secondary structure. Upon close inspection, it became clear that these cleavage sites fall within or are adjacent to the most evolutionarily conserved long-range RNA tertiary interactions that are essential for correctly folding the ribozyme (annotated by Greek letters, Fig. 2b)^41^. To further understand the conformation of these sites in 3-D space, the Tb^3+^ signal was visualized on the crystal structure of *O.i*. intron (Fig. 2a). We found that Tb^3+^ causes backbone cleavage at regions where the phosphate backbone compresses together to form sharp, stable turns. These turns are all components of RNA tertiary motifs required for the correct folding of the active ribozyme.

To examine the sites of cleavage in greater detail, we focus on two regions that are specifically recognized by Tb^3+^. The first is the ζ-ζ’ tetraloop-receptor interaction, which is among the best-characterized and most important interactions for positioning catalytic intron domain 5 (D5)^41^. Here, a single G236 residue in D1 flips out of a sharp backbone turn and base-stacks with A370 in D5 (Fig. 2b top insert). We observe strong Tb-seq signals for the nucleotides in this bulge (234-237) that mediates the ζ-ζ’ tetraloop interaction. The second motif, λ-λ’ is within the z-anchor, a module that forms multiple higher-order structures and serves as a scaffold for properly positioning the 5’ splice site. Notably, strong Tb-seq signal is observed in A106 in D1, which forms a minor groove base triple with nucleotides C267 and G374 in D5 (Fig. 2b bottom insert). These results demonstrate that Tb^3+^ detects functionally important interactions in group II introns where RNA phosphate backbones come into very close proximity, thereby allowing for multi-helix base stacking and long-range interactions.

**Fig. 3:**
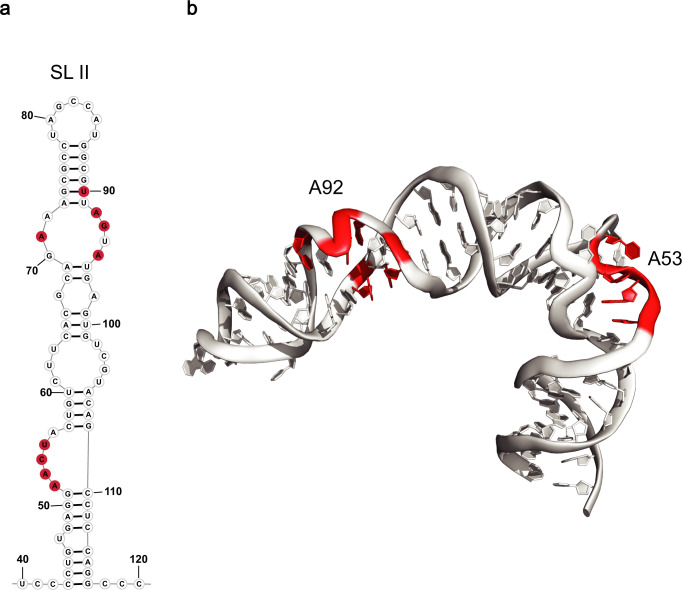
Tb-seq of HCV IRES detects conserved L-shaped bend in stem loop II. **a** Secondary of HCV 5’ UTR Stem loop (SL) II displaying sites of strong Tb^3+^cleavage (red). **b** 3-D structure of SLII showing sites of strong Tb^3+^ cleavage on the RNA backbone (red highlight). Adapted from PDB “5A2Q”. Source data are provided as a Source Data file.

To further test and expand Tb-seq, we probed another class of RNA that contains a well-defined tertiary structure. For this we chose the Hepatitis C. Virus (HCV) internal ribosome entry site (IRES), specifically focusing on domain II, which has well-characterized structural features identified by both cryoEM^42^ and NMR^43^. Implementing the criteria described above, we observed strong Tb^3+^ signal clustering in two regions. The first is a loop region containing nucleotides 92-95, where the phosphate backbones kink and come into close proximity (Fig. 3). The second region is near nucleotides 52-54, where the phosphate backbone forms a nearly 90˚ bend in the RNA (Fig. 3). This bend is implicated in the positioning of the downstream terminal loop near the 40 S E site of the ribosome, which allows for translation of viral proteins^42,44^. Interestingly, this region has been targeted by functional inhibition studies where multiple small molecules bind and structurally extend the bend into an elongated conformation, inhibiting viral translation^45,46^. Together these results indicate terbium probing can detect functionally important structures in RNAs, allowing it to be used as a screening tool for identifying regions that are likely to contain compact motifs.

### Tb-seq detects key RNA-protein interactions in a cellular context

**Fig. 4:**
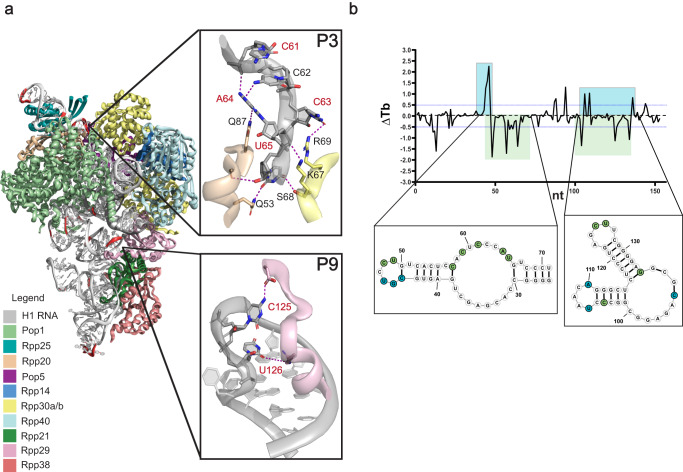
Probing RNA-Protein interactions in human RNase P. **a** 3-D structure of RNase P complexed with its protein components showing sites of strong Tb^3+^ cleavage on the RNA backbone (red highlight). Inserts show close up views of two regions containing RNA-Protein interactions with nucleotides that display Tb^3+^ cleavage labeled (red) and hydrogen bonds (purple dashed lines). Adapted from PDB “6AHR”. **b** Δ Tb reactivity profile for the first 150nt of RNase P. Nucleotides indicated in green become less reactive when probed in the absence of protein components, whereas nucleotides indicated in blue become more reactive. Source data are provided as a Source Data file.

Having established the versatility of Tb-seq on RNAs that have been in-vitro transcribed, we sought to extend it to cellular contexts, where RNA can fold together with proteins, small molecule ligands and other nucleic acids^7^. We decided to conduct the first experiments on a structurally well-defined cellular RNA with known protein binding sites. To this end, we probed human RNase P in order to understand how terbium can be used to reveal higher-order RNA structural motifs in that stable RNP. To circumvent the issues of introducing lanthanide ions into cells, we developed an approach for gently lysing mammalian cells in a way that maintains intact RNA-Protein (RNP) complexes (Supplementary Fig. 5A). We then treated the resulting extract with Tb^3+^ and implemented the Tb-seq pipeline, using the criteria we established for identifying strong sites of specific Tb^3+^ cleavage (Supplementary Fig. 5B). By comparing the Tb-seq signal with the cryo-EM structure of human RNase P H1 RNA^47^, we observe that the strongest cleavage sites are found in regions where the RNA backbone bends sharply, notably at the top and bottom of the H1 RNA (nt 47–50 and 169–173; Fig. 4a).

Human RNase P consists of ten protein components that wrap around and bind the H1 RNA at multiple regions, presumably stabilizing its elongated conformation^47^ (Fig. 4a). While a number of sites are observed, here we highlight two examples where Tb-seq reveals regions containing critical RNA-protein interactions. The first is a backbone turn located in the loop of stem P9 (Fig. 4a, bottom insert). The bases of nucleotides C125 and U126 form hydrogen bonding interactions with the side chains of the essential core protein, Rpp29. This protein makes multiple contacts with stem P9 and P1, bringing them together in close proximity and stabilizing the downstream helical core of the H1 RNA, which recognizes the 5’ end of pre-tRNA for cleavage. The second site of strong Tb-seq signal is observed in the loop region of stem P3. Here, the backbone, bases, and sugars of the nucleotides targeted by Tb^3+^ (C61, C63, A64, U65), form networks of hydrogen bonds with proteins Rpp20 and Rpp30b (Fig. 4a, top insert). In this context, Tb-seq signals correspond to exposed regions of the RNA, which form structural motifs that are stabilized by protein interactions within RNase P.

To further explore the ability of Tb-seq to reveal RNP interactions and to understand the role of the protein in Tb^3+^ detection at these sites, we used Tb^3+^ to probe human RNase P in the absence of proteins. To this end, Tb^3+^ cleavage was conducted on cell lysates that were treated with a proteolytic enzyme (Proteinase-K), which strips proteins from RNA. (Supplementary Fig. 6A). As in studies with other chemical probes^48^, we then performed a differential reactivity comparison, termed ∆ Tb, to compare changes in H1 RNA structure in the presence and absence of proteins (Fig. 4b and Supplementary Fig. 6B). Consistent with a disruption of a stabilizing protein interaction, the two regions described above become less reactive in the absence of proteins (show a loss in terbium reactivity). By contrast, other nucleotides become more reactive after proteinase K treatment (see stem P3, Fig. 4b), which may result from conformational rearrangement that occurs in the absence of proteins. These data suggest that ∆ Tb detects modules of protein-stabilized RNA structures within RNase P, thereby broadening the applicability of this method to probing of RNP interfaces.

### Tb-seq reveals modules of higher-order structure in viral RNAs

Having validated Tb-seq as an RNA tertiary structure probe, we sought to apply it to discover previously unknown RNA structures in multi-kilobase RNAs, such as long viral RNA genomes. Numerous studies have demonstrated that viral RNA genomes contain secondary and tertiary structures both in the UTRs and coding regions that are important for function^49–51^. Indeed, we utilized Tb-seq to detect functional RNA structures within the HCV IRES (Fig. 3). Given the urgency of detecting functional RNA elements within SARS-CoV-2 RNA^49,52^ and the limited tools available to detect them, we performed cell lysate Tb-seq in SARS-CoV-2 infected cells. We specifically examined the 5’-terminal 1400nt of the RNA genome, which contains the 5’UTR, the coding region of Nsp1 and part of the Nsp2 ORF.

**Fig. 5:**
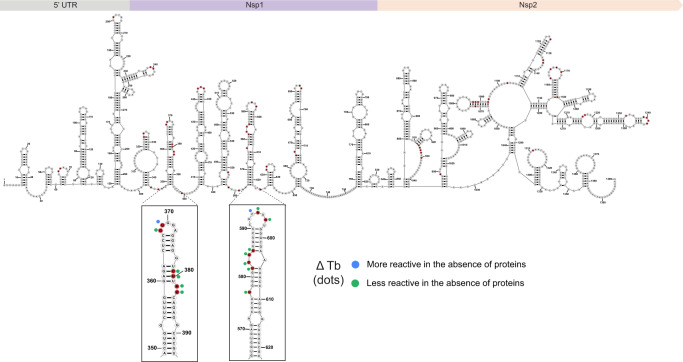
Tb-seq identifies structural modules in SARS-CoV-2. Cell lysate probing of the 5’ terminal of SARS-COV2. Secondary structure of the SARS-CoV-2 displaying sites of strong Tb^3+^cleavage (red). Inserts showing close-up views of two regions that display Δ Tb reactivities. Source data are provided as a Source Data file.

Inspection of the Tb-seq signal profile reveals a distinct cleavage pattern that is characterized by clusters of consecutive cleaved nucleotides (Supplementary Fig. 7). This signal profile resembles that obtained when probing ribozymes, suggesting a high degree of 3-D structure in the genome. Overlaying these sites onto the predicted secondary structure^49^, we observe strong Tb^3+^ signals in both the UTR and coding region of the genome. Upon closer inspection, we find the majority of Tb-seq signals in small stem-loop/bulge regions, implicating these regions as modules of compact RNA structure (Fig. 5).

To further understand the role of protein occupancy on this structured genome and to narrow down sites of potentially functional RNA modules, we probed in the absence of proteins and implemented the ∆ Tb pipeline. Numerous changes are observed in the absence of protein, indicating a global conformational change in the architecture of the genome (Supplementary Fig. 8). At some sites, the reactivity signal increases, implicating a conformational change in RNA tertiary structure or new backbone accessibility in the absence of proteins. By contrast, there are other sites that become less reactive upon the release of proteins (Fig. 5 inserts). Given our findings with probing RNase P, these sites are likely to represent structural modules containing a sharp backbone bend that is stabilized by protein components. The limited proteomic information on the SARS-CoV-2 genome makes it difficult to assess specific interaction partners. Nevertheless, together these data underscore the utility of combinatorial Tb-seq for narrowing down structural modules and providing a course-grained roadmap of candidate functional elements within a viral genome.

## Discussion

As biologists explore the growing landscape of biologically important multi-kilobase RNAs, such as viral genomes, unprocessed mRNAs, primary miRNAs and long noncoding RNAs, tools are needed that will enable researchers to focus their attention on specific regions of RNA for detailed functional analysis. The Tb-seq pipeline presented here provides one such filter, yielding valuable information about structurally compact local RNA motifs that differs from the information reflected in other probes of secondary and tertiary structure. In addition, by using the ∆ Tb probing strategy, and probing in the presence and absence of protein components, one can narrow down tertiary structures that undergo protein-dependent conformational differences. Ultimately, integrating Tb-seq with orthogonal chemical probes, pull-down methods^53,54^, cross-linking agents^20,21^ and functional assays will allow for a comprehensive mechanistic understanding of individual RNA molecules.

With recent technological advances, it is now possible to determine high-resolution structures of large RNAs^55^. However, multi-kilobase RNAs cannot be visualized in their entirety using these approaches. Most biologically relevant transcripts contain modules of compact structure along with regions that are conformationally flexible^56–58^. For this reason, most RNAs are amenable to high-resolution structure determination only after careful study of their overall structural landscape. This requires a methodical approach for identifying RNA regions and RNP substructures that can be visualized with powerful tools such as cryo-EM and SAXS. In addition, there are many cases where one must rationally design or isolate stable motifs of RNA and/or RNP complexes. Here we provide a way to identify the most structurally compact regions of a large RNA and, in tandem with other long-range probing methods, choose the best regions for high-resolution investigation.

Performing Tb-seq on RNAs with known structures provided a useful starting point for assessing the types of RNA motifs that are recognized and cleaved by Tb^3+^. We initially attempted to correlate Tb-seq signals with specific torsion angles and with atomic distance vectors between different base and backbone atoms, but no clear correlation emerged. In order to develop a less fine-grained structural correlation metric, we visualized the structures and noted that most Tb^3+^ cleavage sites occur in regions where multiple phosphate backbone residues pinch together in close proximity. To reflect this, we computed a metric for assessing the “sharpness” of turns in the RNA backbone at Tb^3+^ cleavage sites, deriving our values from high-resolution structures of the *O.i*. intron^39^. Specifically, we measured the backbone phosphate distances between nucleotide n to nucleotide n + 2 at sites displaying strong Tb-seq signals (P_n_ -> P_n+2_, or every other phosphate) and compared these data to the corresponding distances in a simple helical structure within domain 4 of the intron (Supplementary Fig. 9). We found that RNA regions with strong Tb-seq signals tend to have very small P_n_ -> P_n+2_ values (5.5 - 9.1 Å) relative to the same distances calculated from a simple helix (9.6-12.5 Å), indicating local compression of the RNA backbone. We speculate that additional data will allow for the refinement of these parameters. For example, while some Tb^3+^ cleavage sites, such as those in region 7, are not characterized by small P_n_ -> P_n+2_ values, visual inspection of the structure shows that these same nucleotides are part of a larger motif in 3-D space that contains adjacent pinched backbones that are characterized by strong Tb-seq signatures and small P_n_ -> P_n+2_ values (in region 5). Therefore, the same bound metal ion may be catalyzing both cleavage events. Additional data and analysis will enable a more quantitative description of specific structural features that are recognized and cleaved by Tb^3+^.

There are limitations in our ability to interpret the structural significance of Tb^3+^ cleavage signals in RNA probing studies. First of all, metal ions like Tb^3+^ cannot stimulate the cleavage of an RNA backbone unless the 2’-hydroxyl nucleophile adopts a precisely in-line geometry capable of phosphodiester cleavage. Certain architectural environments may constrain local structure such that in-line attack is not possible, and it is therefore likely that some Tb^3+^-bound sites remain uncleaved by the probe, resulting in false-negative data. Furthermore, it is not possible to interpret a Tb^3+^ cleavage site as reflective of a particular type of RNA structural motif. This is because there is simply not enough information on site-bound metal ions in the available RNA structural database. Although it is hoped that this may grow with time, the emergence of RNA cryo-EM structure determination (which cannot unambiguously assign metal ion sites) and the decline in the prevalence of crystallographically-determined structures (where metals can be explicitly assigned), indicates that progress will be slow, making it all the more important that additional orthogonal information on metal sites in RNA is obtained using other methods. As discussed in the previous paragraph, additional data will be necessary to understand the precise structural features that result in cleavage by Tb^3+^. That said, it is reasonable to claim that Tb^3+^ cleavages reflect metal ion binding to sites of high electronegative potential that result from the close-packed RNA backbone atoms that are prevalent within RNA tertiary structures and RNP complexes.

The human transcriptome contains a vast set of large, complex RNA molecules, and until recently, we have lacked the tools to assess their 3-D structural content. However, the biochemical methods that were initially developed to study tRNAs, riboswitches and ribozymes are being gradually being adapted to explore the growing repertoire of multi-kilobase RNAs that are central to gene expression and pathogenicity. Here we present a much-needed expansion of the RNA probing toolbox that allows investigators to rapidly pinpoint candidate RNA tertiary structures efficiently and precisely, paving the way for downstream mechanistic study and therapeutic targeting.

## Methods

### In vitro transcription and purification

The in vitro transcriptions of aI5γ D135^59^ and *Oceanobacillus iheyensis (O.i.)* group II intron D1-5^39^, RNA were carried using T7 RNA polymerase^60^ in a buffer containing 12 mM MgCl_2_, 40 mM Tris-Cl pH8, 2 mM Spermidine, 10 mM NaCl, 0.01% Triton X-100, 10 mM DTT, 5 μl SUPERase-In and 3.6 mM of each NTP, in a procedure adapted from previous work^61^. The reactions were incubated at 37 °C for 2 hours. Thereafter, 4U of TURBO DNase was added and the mixture was incubated at 37 °C for 30 min. To chelate excess divalent ions, 5 μl of 0.5 M EDTA was added. Transcription products were gel purified on a denaturing 5% polyacrylamide gel and eluted overnight at 4 °C in a gel elution buffer (10 mM MOPS-NaOH pH 6.0, 300 mM NaCl and 1 mM EDTA). The RNA was ethanol precipitated and resuspended in ME buffer (6 mM K-MES pH 6.0, 0.1 mM EDTA). The in vitro transcription of the full-length HCV genome (JC1)^51,62^ was performed as described above. The transcribed RNA was buffer exchanged into a filtration buffer (50 mM HEPES-KOH pH7.2 and 150 mM KCl using 50-kDa Amicon Ultra filtration columns. The RNA was purified by size exclusion chromatography at room temperature using a self-packed 24 ml Sephacryl S-1000 column equilibrated with filtration buffer. RNA from the peak fraction was used for subsequent folding and probing.

### RNA folding and Tb^3+^ probing

For D135, Tb^3+^ cleavage was performed using two approaches. The first was direct visualization of Tb^3+^ mediated RNA cleavage by electrophoresis^36^. In-vitro transcribed D135 was dephosphorylated using Antarctic phosphatase and 5′ end-labeled with [γ-^32^P] ATP using T4 polynucleotide kinase according to manufacturer’s instructions followed by purification on a denaturing 5% polyacrylamide gel. Thereafter, 3 nM of ^32^P-labeled RNA and 1 μg of unlabeled RNA were mixed in a monovalent buffer containing 50 mM MOPS pH7 and 500 mM KCl to a final volume of 18 μl. For visualization of Tb^3+^ mediated cleavage sites by reverse transcription and sequencing 1 μg of unlabeled RNA was used. For all reactions, the mixture was heated up to 90 °C for 1 min and cooled at room temperature for 2 min. Thereafter, 2 μl of 1 M MgCl_2_ (final concentration 100 mM) was added and folded at 37 °C for 30 min. Subsequently, probing was performed by incubating 18 μl of the folded RNA with 10X TbCl_3_ stocks prepared in the monovalent buffer (final 1x concentration from 0.01mM-2mM TbCl_3_) or 2 μl of monovalent buffer (negative control) for 40 min at 25 °C. For the time-course experiments, probing was performed at the indicated times. All reactions were quenched with the addition of 3 μl of 50 mM EDTA pH 8 and precipitated by adding 1/10 volume of Na-Acetate (3 M, pH 5.2), 0.5 μl of glycogen (Invitrogen) and three volumes of ethanol. RNAs were resuspended in 4 μl of loading buffer (82 % (v/v) deionized formamide, 0.16 % (w/v) xylene cyanol (XC), 0.16 % (w/v) bromophenol blue (BB), 10 mM EDTA, pH 8.0) and resolved on a denaturing 5% polyacrylamide gel. The gel was dried, exposed to phosphor screens overnight and scanned using a Typhoon FLA9500 phosphorimager (GE Healthcare) or Typhoon RGB Biomolecular imager (Cytiva).

For Tb^3+^ probing of *O.i*. and HCV, 1 μg of RNA was diluted in their respective monovalent ion buffers (50 mM HEPES pH7 and 150 mM KCl for *O.i*. or 50 mM HEPES pH7.2 and 150 mM KCl for HCV) to a final volume of 18 μl. Thereafter, 2 μl of 100 mM MgCl_2_ (final concentration 10 mM) was added and incubated at 37 °C for 30 min. Subsequently, probing was performed by incubating 18 μl of the folded RNA with 2 μl of 10X TbCl_3_ stocks prepared in their respective monovalent ion buffers (final 1X concentration from 0.01mM-2mM) or 2 μl of respective monovalent buffer (negative control) for 10 min at 25 °C. Reactivities were compared under conditions where 0.5 mM TbCl_3_ was employed and used in all figures unless indicated otherwise. All reactions were quenched with the addition of 3 μl of 50 mM EDTA pH 8. For the denaturing control, RNA was folded as described above but afterward, deionized formamide was added to a final concentration of 50%. The denatured RNA was probed with a final concentration of 0.5 mM TbCl_3_. For the secondary structure control, the RNA was incubated in a monovalent buffer in the absence of MgCl_2_ and probing was carried out at the indicated TbCl_3_ concentrations. All RNA samples were cleaned up using a Zymo RNA clean and concentrator column according to the manufacturer’s instructions.

#### Native gel electrophoresis

Radiolabeled transcripts^40^ were prepared as described above except, 1 mM UTP and 50 μCi of [α-^32^P-UTP] were added to the transcription. The reaction was incubated at 37 °C for 2 h followed by purification on a denaturing 5% polyacrylamide gel. 5 nM of the radiolabeled transcript was spiked into the folding reaction described above. Reactions were mixed with 10% (v/v) glycerol containing, 0.16 % (w/v) xylene cyanol, 0.16 % (w/v) bromophenol blue and loaded onto a native 5% polyacrylamide gel.

### Cell culture of human RNase P and SARS-CoV-2 infection

For in-cell studies of RNase P RNA structure, Huh7.5 cells (Cells were a gift from Brett Lindenbach. Cell line was generated as described in{Blight, 2002 #112}) were cultured in Dulbecco’s Modified Eagle Medium (DMEM w/o sodium pyruvate) that was supplemented with 10% heat-inactivated fetal bovine serum (FBS) and 1 mM non-essential amino acids. Cells were cultured to ~80% confluency (~5 × 10^6^ cells) in a 150 cm tissue culture-treated dish.

For studies of SARS-CoV-2 RNA, Huh7.5 cells were cultured in DMEM supplemented with 10% FBS and 1% Penn/Strep. Approximately 5 × 10^6^ cells were plated in each of the T150 tissue culture-treated flasks and incubated overnight at 37 °C/5% CO_2_. The next day, media was removed and 5 × 10^5^ PFU (MOI ~ 0.1) of SARS-Related Coronavirus 2 Isolate USA/WA2020 (BEI Resources #NR-52281) was added to each flask in fresh media. Cells were incubated with virus inocula until three days post-infection (dpi).

### Cell lysis probing

For all flasks the media was aspirated, cells were washed once with cold wash buffer (50 mM HEPES-KOH pH7.2, 150 mM NaCl, 3 mM KCl), and then dislodged in 2 ml of cold wash buffer with a cell scraper. The cells were collected and centrifuged at 200 g x 5 min at 4 °C. The supernatant was removed and the cells were resuspended in 2 ml lysis buffer (1% TritonX-100 50 mM HEPES-KOH, pH7.2, 150 mM KCl, 18 mM NaCl, 1 mM MgCl_2_, 1 mM CaCl_2_, 30ul SUPERase-In (20U/μl) and 1x cOmplete Protease Inhibitor Cocktail EDTA-free. To 250 μl of resuspended cells, 50/μl of Turbo DNase (2U/μl) was added and the mixture was incubated at 37 °C for 20 min. For cell lysis+Proteinase-K probing experiments, cells were prepared, lysed and DNase digested as described above, but the lysis buffer did not contain protease inhibitor. Subsequently, 25 μl of 20 mg/ml Proteinase-K was added to each 250 μl of lysed cells and the mixture was incubated at 37 °C for an additional 20 min.

All reactions were centrifuged at 200 g x 15 sec. Probing was performed by incubating 225 μl of supernatant with 25 μl of freshly made 10x TbCl_3_ (final 1x concentrations from 0mM-5mM, prepared in wash buffer). The reactions were immediately placed on a rocker and allowed to incubate at 25 °C for 10 min before quenching with 20 μl of 0.1 M EDTA. RNA was extracted using Trizol according to the manufacturer’s instructions. For experiments involving RNase P, total RNA was ribosome depleted using a Ribominus kit that was used according to the manufacturer’s protocol with the following exception: the ribodepleted supernatant was purified using a Zymo RNA clean and a concentrator to retain RNAs that are greater than 17 nucleotides in size. For experiments involving SARS-CoV-2, total RNA was cleaned using a Zymo RNA clean and concentrator column.

### Reverse transcription (RT)

For each probing condition, 1-4 μg of in vitro transcribed or cellular RNA was mixed with 1-2pmol of gene-specific primers (Supplementary Table 1) and brought to a volume of 7 μl. To anneal primers, the mixture was heated at 90 °C for 1 min followed by 30 °C for 2 min. To initiate reverse transcription, 2ul of Marathon RT^37^ (can be obtained from Kerafast), 10 μl of 2x MarathonRT buffer (100 mM Tris-HCl pH 8.3, 400 mM KCl, 4 mM MgCl2, 10 mM DTT and 40% glycerol), 1 μl of 10 mM dNTP mix (NEB) were added and incubated at 42 °C for 30 min. RNA was degraded with the addition of 1 μl of 3 M KOH, heated to 95 °C for 5 min and snap cooled to 4 °C for 5 min. Thereafter, 1 μl of 3 M HCl was added to neutralize the reaction. For primer extension reactions that would be visualized using electrophoresis, reverse transcription was carried out as described, but using a ^32^P-labeled primer. The primer was labeled at the 5-end using T4 PNK according to manufactures instructions and purified on a denaturing 12% polyacrylamide gel. After reverse transcription, the cDNA was ethanol precipitated at −20 °C overnight. The cDNA pellets were dissolved in 5 μl of loading buffer (82% deionized formamide, 10 mM EDTA pH 8, 0.2% xylene cyanol and bromophenol blue) and resolved on a denaturing 5% polyacrylamide gel. The gel was dried, exposed to phosphor screens overnight and scanned using a Typhoon RGB Biomolecular imager (Cytiva). For ladder generation, RT was carried out using a Thermo Sequenase cycling kit according to the manufacturer’s instructions with an input of 500 ng of the template.

### Sequencing library preparation

The cDNA products from reverse transcription were purified using AMPure XP beads by adding a 1.2x bead to sample ratio and incubating at room temperature for 10 min. The beads were captured using a magnetic rack for 5 min and washed 3 times with 180 μl of fresh 80% ETOH. The beads were air-dried for 5 min and resuspended in 12 μl of water to elute the cDNA. Thereafter, 3’ adaptor ligation was performed by mixing 8 μl of purified cDNA with 0.2 μl of 50 μM 3’ adaptor (Supplementary Table 1), 1 μl of T4 RNA ligase (NEB), 1 μl of 10 mM ATP, 2 μl of T4 RNA Ligase buffer and8μl of 50% PEG 8000. To reduce ligation bias and barcode the RNA, the ligating adapter contained a random hexamer (NNNNNN) at the 5’ end. The mixture was incubated at 25 °C for 16 h, followed by enzyme deactivation at 65 °C for 15 min. Ligated products were purified with AMPure XP beads using a 1.2x bead to sample ratio. The products were PCR amplified 4-12 cycles with Q5 HF DNA polymerase using Illumina TruSeq forward primer and indexed reverse primers (NEB Next Multiplex Oligos) (Supplementary Table 1), with cycle times of 98 °C for 10 sec, 62 °C for 45 sec, and 72 °C for 60 sec. PCR products were purified with 1.2x volume of AMPure XP beads. Library concentrations were determined using a Qubit dsDNA HS Assay Kit and a BioAnalyzer High Sensitivity DNA Analysis. Libraries were diluted, pooled and sequenced using a NextSeq 500/550 or NextSeq 2000 platform.

### Tb-seq data analysis

All FASTQ files were processed using Cutadapt (v1.9.1) to remove Illumina adapter sequences and then aligned to the respective RNA sequence using HISAT2 (v2.10). Stop information was extracted using RTEventsCounter.py script^38^. The probability of stop per nucleotide was calculated as the number of stops divided by the sum of the total number of read-through events plus the number of stops (Eq. (1)). Probabilities were background subtracted against a no-probe control (Eq. (2)). Only nucleotides that contained more than 10,000 read-throughs were considered. To better compare probing experiments conducted in different contexts, including in vitro and in cell conditions where efficiencies of cleavage might differ, values were normalized to the top 10^th^ percentile of stop rates, then scaled from 1-8 (termed “reactivity,” below, based on^63^).

1 $$P({stop})=\frac{{n}_{{stop}}}{{n}_{{stop}}+{n}_{{read}-{through}}}$$

2 $${Reactivity}={P({stop})}_{{treated}}-{P({stop})}_{{untreated}}$$

Reactivities were compared under conditions where 0.5 mM TbCl_3_ (in-vitro transcribed RNAs) or 1 mM TbCl_3_ (cell lysate RNAs) was employed and used in all figures unless indicated otherwise. For the Δ Tb analysis, the reactivity obtained from cell lysate + Proteinase-K probing experiments was subtracted from the reactivity obtained from cell lysate probing experiments (Eq. (3)). In order to take a conservative approach, a stringent cutoff of +/−0.5 was implemented to detect strong differences in reactivities.

3 $$\Delta {{{{{\rm{Tb}}}}}}={{Reactivity}}_{{Cell}{lysate}+{Proteinase}-K}-{{Reactivity}}_{{Cell}{lysate}}$$

### Structure and graphical display

All secondary structures were visualized and drawn using Structure Editor, v1.0^64,65^. All three-dimensional structure renderings were done using PyMOL Molecular Graphics System, v1.2r3pre, Schrödinger, LLC. Graphical displays were made using GraphPad Prism 8 or RStudio Version 1.2.5001. All gels were visualized using ImageJ 1.52a or ImageQuant TL v8.2.0.0.

### Statistics and reproducibility

Sample size of as *n* = 2 was chosen for most experiments. A sample size in this case corresponds to biological replicates undergoing of full chemical probing data on intact RNAs. No statistical method was used to predetermine sample size. No data were excluded from the analyses. The experiments were not randomized. The investigators were not blinded to allocation during experiments and outcome assessment.

### Reporting summary

Further information on research design is available in the Nature Portfolio Reporting Summary linked to this article.

## Supplementary information

Supplementary Information File

Reporting Summary

## Data Availability

All FASTQ files generated in this study have been deposited in the SRA database under accession code “PRJNA966800”. The processed data generated in this study are provided in the Supplementary Information/Source Data file. The structures used in this study can be found under the accession codes “4E8M”, “5A2Q” and “6AHR”. Source data are provided with this paper.

## Source data

Source Data

## Code availability

All source codes used in this study are available at a GitHub repository: https://github.com/pylelab/Tb-seq.
