# Supplementary material for: Systematic detection of tertiary structural modules in large RNAs and RNP interfaces by Tb-seq
Source: Nat Commun. 2023 Jun 9;14:3426. doi: 10.1038/s41467-023-38623-1 (PMC10255950; doi:10.1038/s41467-023-38623-1)
Supplement: Supplementary file 1 — Supplementary Information File [file 41467_2023_38623_MOESM1_ESM.docx]

**Systematic detection of tertiary structural modules in large RNAs and RNP interfaces by Tb-seq**

Shivali Patel^1^, Alec N. Sexton^1^, Madison S. Strine^2,3^, Craig B. Wilen^2,3^, Matthew D. Simon^1,4^ and Anna Marie Pyle^5,6,7^***

^1^Department of Molecular Biophysics and Biochemistry, Yale University, New Haven, CT, USA

^2^Department of Laboratory Medicine, Yale School of Medicine, New Haven, CT, USA

^3^Department of Immunobiology, Yale School of Medicine, New Haven, CT, USA

^4^Chemical Biology Institute, Yale University, West Haven, CT, USA

^5^Howard Hughes Medical Institute, Chevy Chase, MD, USA

^6^Department of Chemistry, Yale University, New Haven, CT, USA

^7^Department of Molecular, Cellular, and Developmental Biology, Yale University, New Haven, CT, USA

* To whom correspondence should be addressed. Email: [anna.pyle@yale.edu](mailto:anna.pyle@yale.edu)

**Supplementary Table 1:** Reverse transcription primers and DNA oligos used in this study.

| Sequence | Description |
| --- | --- |
| 5’-CAGACGTGTGCTCTTCCGATCTTATCACCTATAGTATAAGT-3’ | Gene specific RT primer for D135 intron. Contains TruSeq overhang. |
| 5’-CAGACGTGTGCTCTTCCGATCTATACGGCGGTTCAAGCTTAGG-3’ | Gene specific RT primer for *O.i*. intron. Contains TruSeq overhang. |
| 5’CAGACGTGTGCTCTTCCGATCTGGAGGAGAGTAGTCTGAATTGGG -3’ | Gene specific RT primer for human RNase P. Contains TruSeq overhang. |
| 5’-CAGACGTGTGCTCTTCCGATCTTTTTCTTTGAGGTTTAGG-3’ | Gene specific RT primer for HCV RNA. Contains TruSeq overhang. |
| 5’-CAGACGTGTGCTCTTCCGATCTCCTGTAAAACAGGCAAACTGAGTTG-3’ | 1 of 5 gene specific RT primers for SARS-CoV-2. Contains TruSeq overhang. |
| 5’-CAGACGTGTGCTCTTCCGATCTCCGTACTGAATGCCTTCGAG-3’ | 2 of 5 gene specific RT primers for SARS-CoV-2. Contains TruSeq overhang. |
| 5’-CAGACGTGTGCTCTTCCGATCTTAATGCACTCAAGAGGGTAGC-3’ | 3 of 5 gene specific RT primers for SARS-CoV-2. Contains TruSeq overhang. |
| 5’-CAGACGTGTGCTCTTCCGATCTGGTGTCAAATTTCTTTGCC-3’ | 4 of 5 gene specific RT primers for SARS-CoV-2. Contains TruSeq overhang. |
| 5’-CAGACGTGTGCTCTTCCGATCTCAAGACTATGCTCAGGTCC-3’ | 5 of 5 gene specific RT primers for SARS-CoV-2. Contains TruSeq overhang. |
| 5’Phos-NNNNNNAGATCGGAAGAGCGTCGTGTAG-3’Bio | DNA oligo used to ligate 3’-end of cDNAs. |

**Supplementary Table 2:** Reagents used in this study.

| MgCl_2_ | Sigma-Aldrich, S255777 |
| --- | --- |
| NaCl | Sigma-Aldrich, S9888 |
| KCl | Sigma-Aldrich, 409316 |
| CaCl_2_ | Sigma-Aldrich, 21097 |
| TbCl_3_ | Sigma-Aldrich, 212903 |
| Spermidine | Sigma-Aldrich, 85558 |
| EDTA | Thermo-Fisher, AM9260G |
| Tris buffer | Thermo-Fisher, 15504020 |
| MOPS | Sigma-Aldrich, M1254 |
| HEPES | Sigma-Aldrich, H7006 |
| MES | Sigma-Aldrich, M0895 |
| Sodium Acetate | Sigma-Aldrich, S3889 |
| Triton X-100 | Sigma-Aldrich, T9284 |
| DTT | GoldBio, 27565-41-9 |
| Adenosine 5'-triphosphate (sodium salt) | Cayman Chemicals, 14498 |
| Uridine 5'-triphosphate (sodium salt) | Cayman Chemicals, 9003530 |
| Guanosine 5'-triphosphate (sodium salt) | Cayman Chemicals, 16060 |
| Cytidine 5'-triphosphate (sodium salt) | Cayman Chemicals, 18147 |
| SUPERase-In | Thermo-Fisher, AM2694 |
| TURBO DNase | Thermo-Fisher, AM2238 |
| Proteinase-K | Thermo-Fisher, AM2548 |
| RNA grade Glycogen | Thermo-Fisher, R0551 |
| Formamide, Deionized | Sigma-Aldrich, S4117 |
| 50-kDa Amicon Ultra filtration columns | Millipore, UFC510096 |
| cOmplete Protease Inhibitor Cocktail EDTA-free | Roche, 05056489001 |
| [γ-^32^P] ATP | PerkinElmer, NEG035C001MC |
| [a-^32^P] ATP | Perkin Elmer, BLU007H250UC |
| Thermo Sequenase cycling kit | Thermo-Fisher, 785001KT |
| Antarctic phosphatase | NEB, M0289S |
| T4 PNK | NEB, M0201S |
| dNTP | NEB, N0447L |
| T4 RNA ligase 1 | NEB, M0204S |
| Q5 HF DNA polymerase | NEB, M0491S |
| NEB Next Multiplex Oligos Sets 1-4 | NEB, E7335S, E7500S, E7710S, E7730S |
| AMPure beads | Beckman coulter, A63880 |
| Trizol | Thermo-Fisher, 15596026 |
| Chloroform: Isoamyl alcohol 24:1 | Millipore-Sigma, C0459-1PT |
| DPBS | Genesee, 25-508 |
| DMEM w/o sodium pyruvate | Thermo-Fisher, 11965118 |
| Fetal Bovine Serum (FBS) | Genesee, 25-550H |
| Non-essential amino acids | Thermo-Fisher, 11140050 |
| Penicillin-Streptomycin | Thermo-Fisher, 15070063 |
| RiboMinus Human/Mouse Transcriptome Isolation Kit | Invitrogen, 155001 |
| RNA clean and concentrator | Genesee, R1013 |
| Qubit dsDNA HS Assay Kit | Thermo-Fisher, Q32851 |
| BioAnalyzer High Sensitivity DNA Kit | Agilent, 5067-4626 |
| NextSeq 500/550 Mid Output kit v2.5 (150 cycles) | Illumina, 20024904 |
| NextSeq 1000/2000 P1 Reagents (300 Cycles) | Illumina, 20050264 |

**
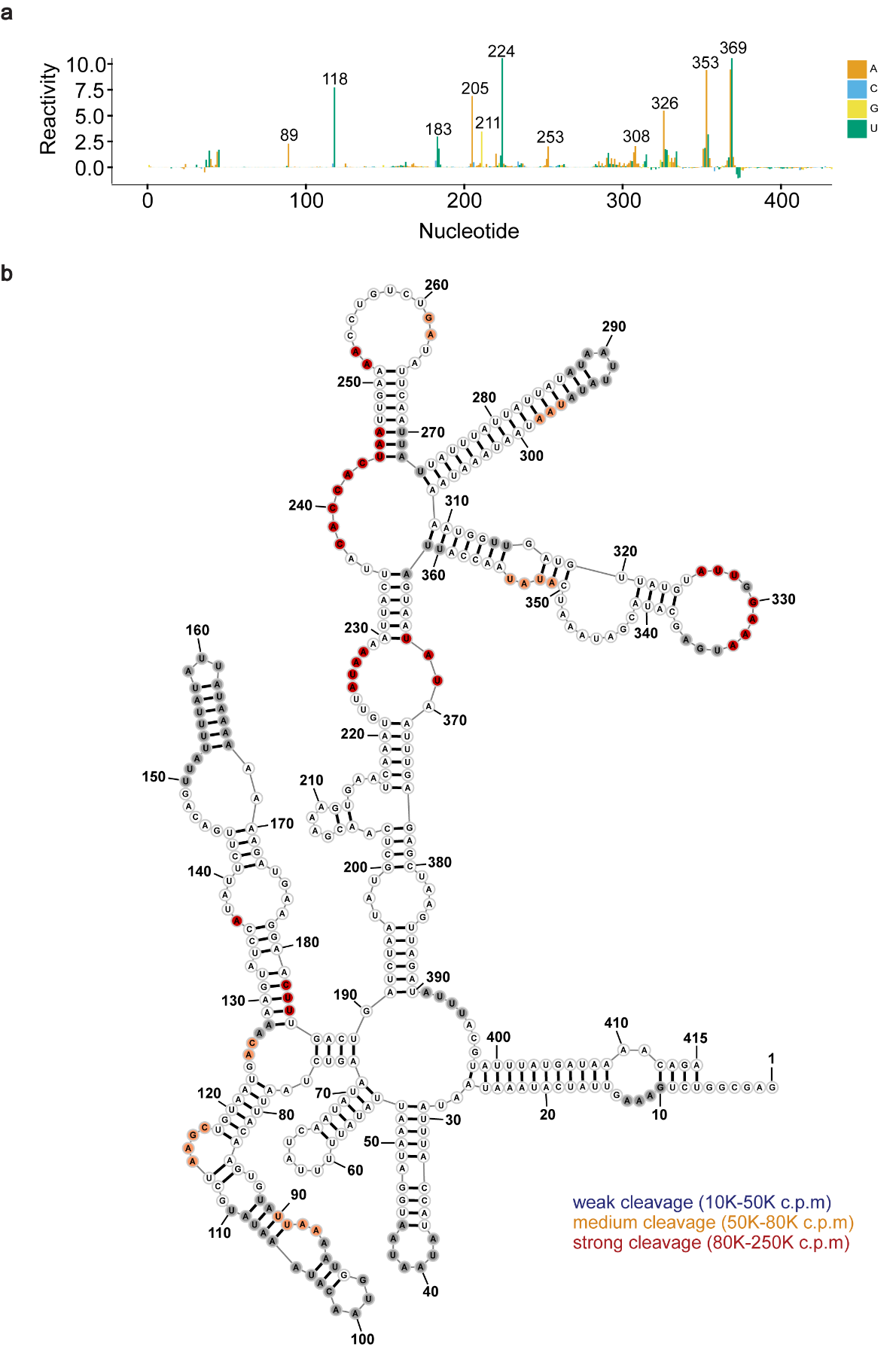
**

**Supplementary Figure 1.** Tb-seq on D135 recapitulates previously reported cleavage sites.

1. Bar plot showing reactivity profile obtained when probing D135. Source data are provided as a Source Data file.
2. Secondary structure of D135 domain I with Tb^3+^ cleavage sites determined from electrophoresis^1^.

**
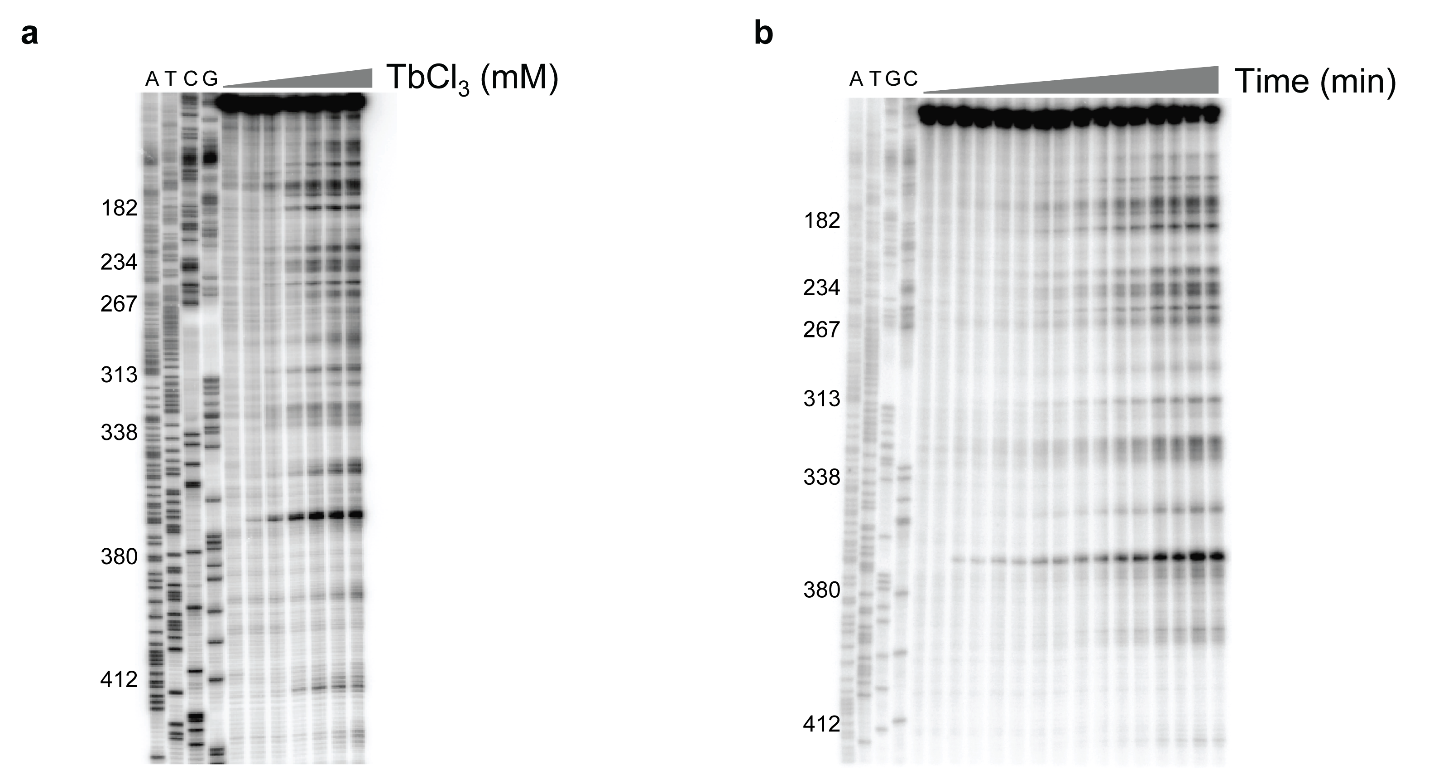
Supplementary Figure 2.** Optimizing Tb^3+^ probing conditions.

1. Primer extension gel of D135 Tb^3+^ probed for 10 min at the indicated concentrations. The TbCl_3_ concentrations are 0, 0.01, 0.1, 0.25, 0.5, 1, and 2 mM. N=2.
2. Time resolved primer extension gel of D135 probed at 0.5mM TbCl_3_. Times of probing are: 0, 0.1, 0.2 0.5, 1, 2, 3, 5, 7, 10, 15, 20, 30, 45, 60, and 90 min. N=2.

**
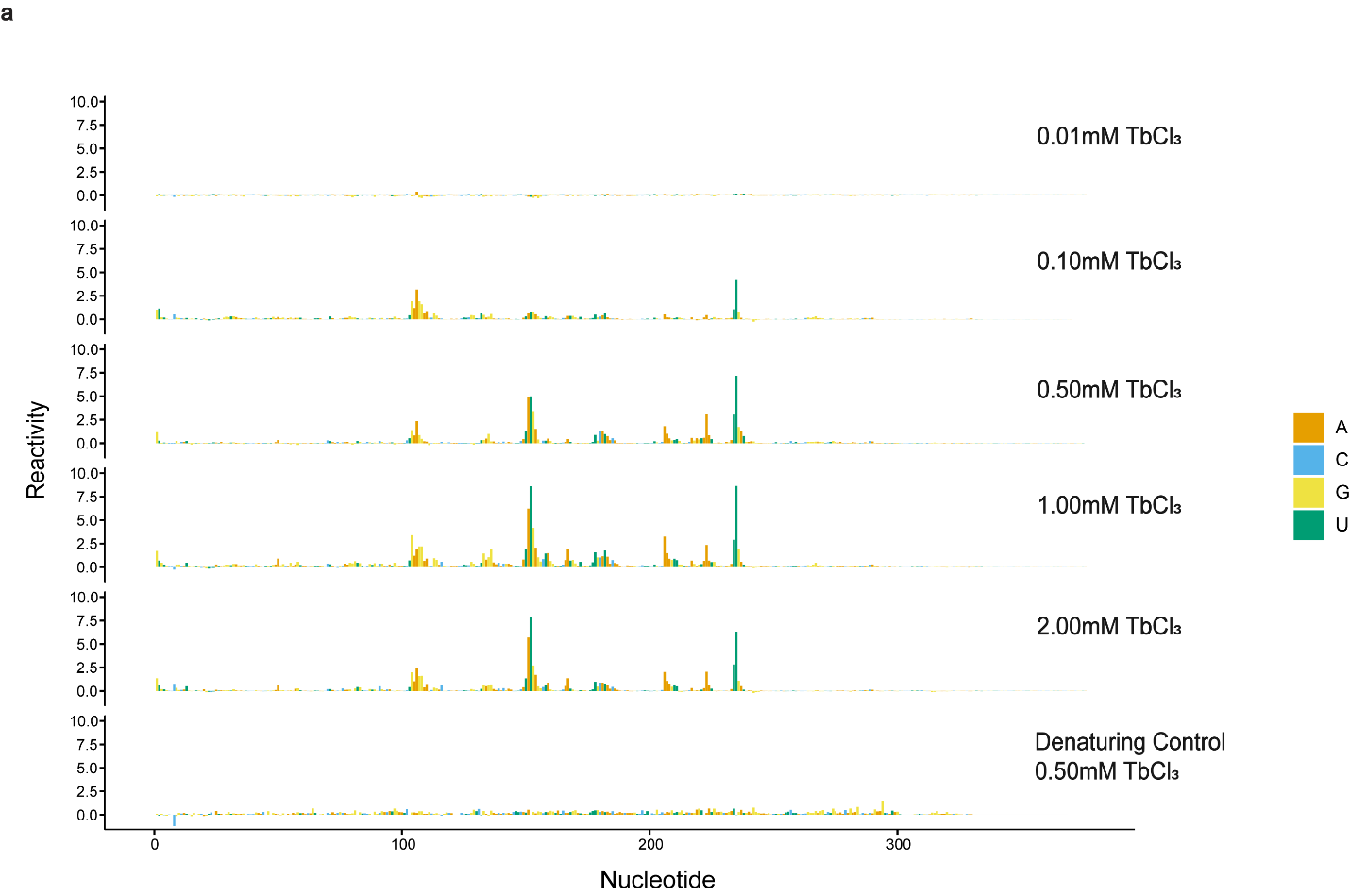
**

**Supplementary Figure 3.** Tb-seq reactivity profile of *O.i.* intron.

1. Bar plot showing reactivity profile obtained when Tb^3+^ probing *O.i.* intron at the indicated concentrations. Source data are provided as a Source Data file.


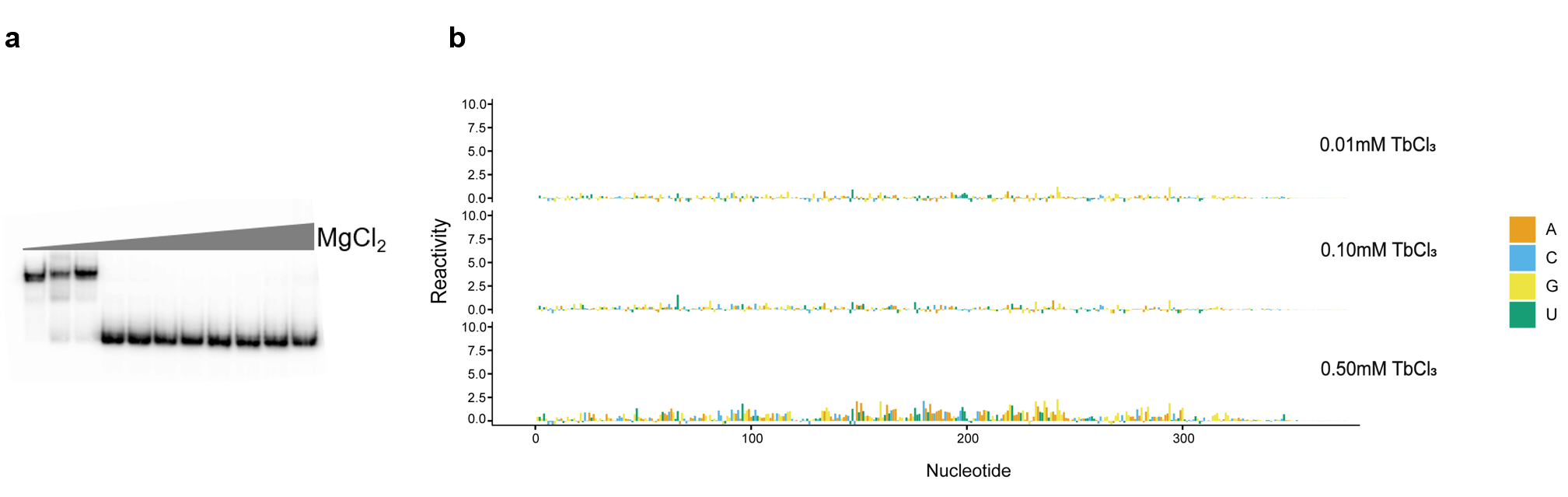


**Supplementary Figure 4.** Tb^3+^ probing the secondary structure of *O.i.* intron does not result in a distinct cleavage pattern.

1. Electrophoretic mobility shift assay of *O.i.* intron. The MgCl_2_ concentrations are 0, 0.1, 0.5, 1, 2, 5, 10, 15, 20, 30 and 50 mM. N=2. Source data are provided as a Source Data file.
2. Bar plot displaying reactivity values when probing *O.i.* intron containing only secondary structure. Source data are provided as a Source Data file.


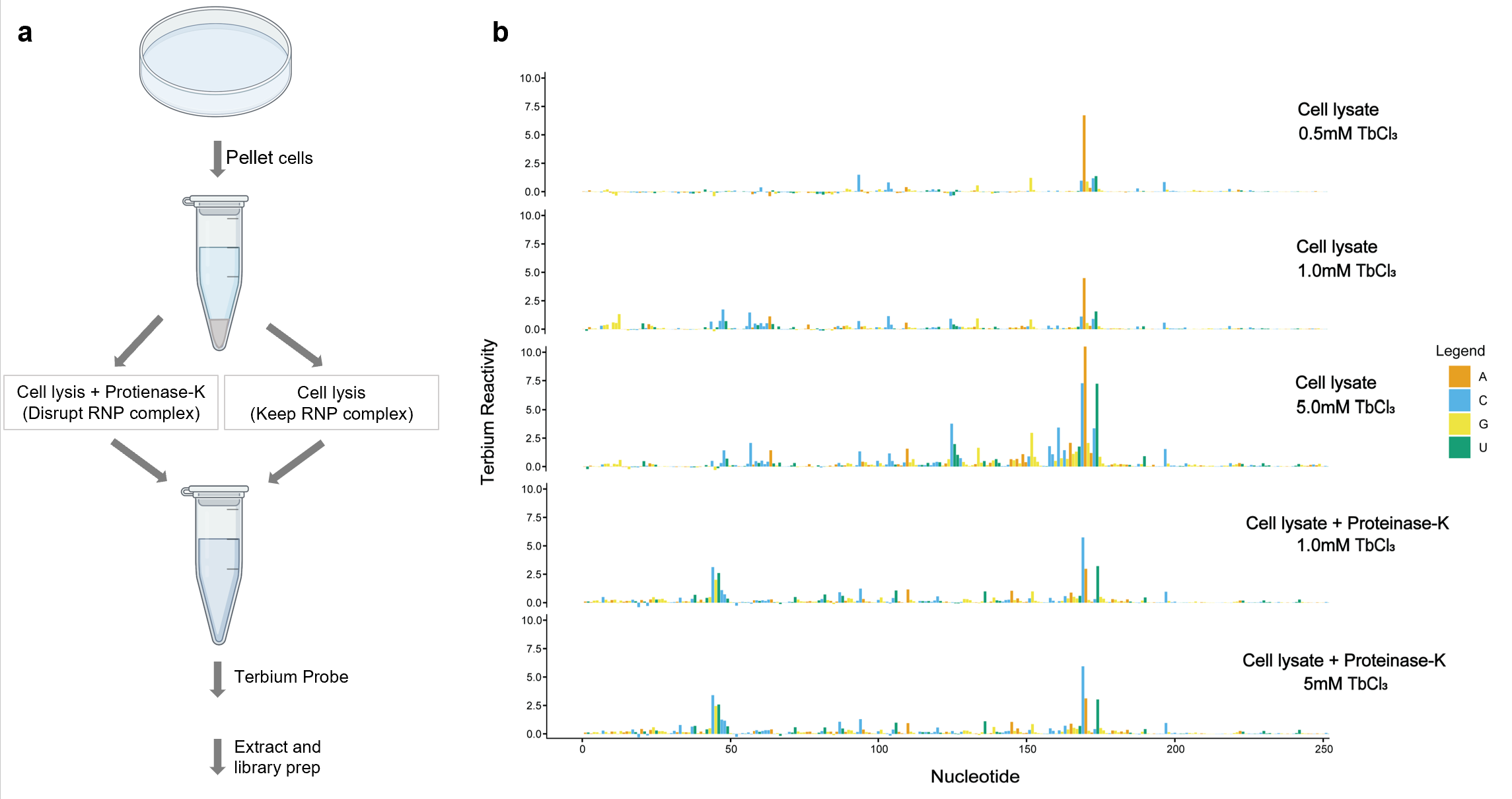


**Supplementary Figure 5.** Establishing cell lysis Tb^3+^ probing on human RNase P in the presence and absence of proteins.

1. Schematic displaying overview of cell lysis probing to either retain or disrupt RNA-protein (RNP) complexes. Created with BioRender.com
2. Bar plot displaying Tb^3+^ reactivity values when probed at the indicated concentrations and probing conditions. Source data are provided as a Source Data file.


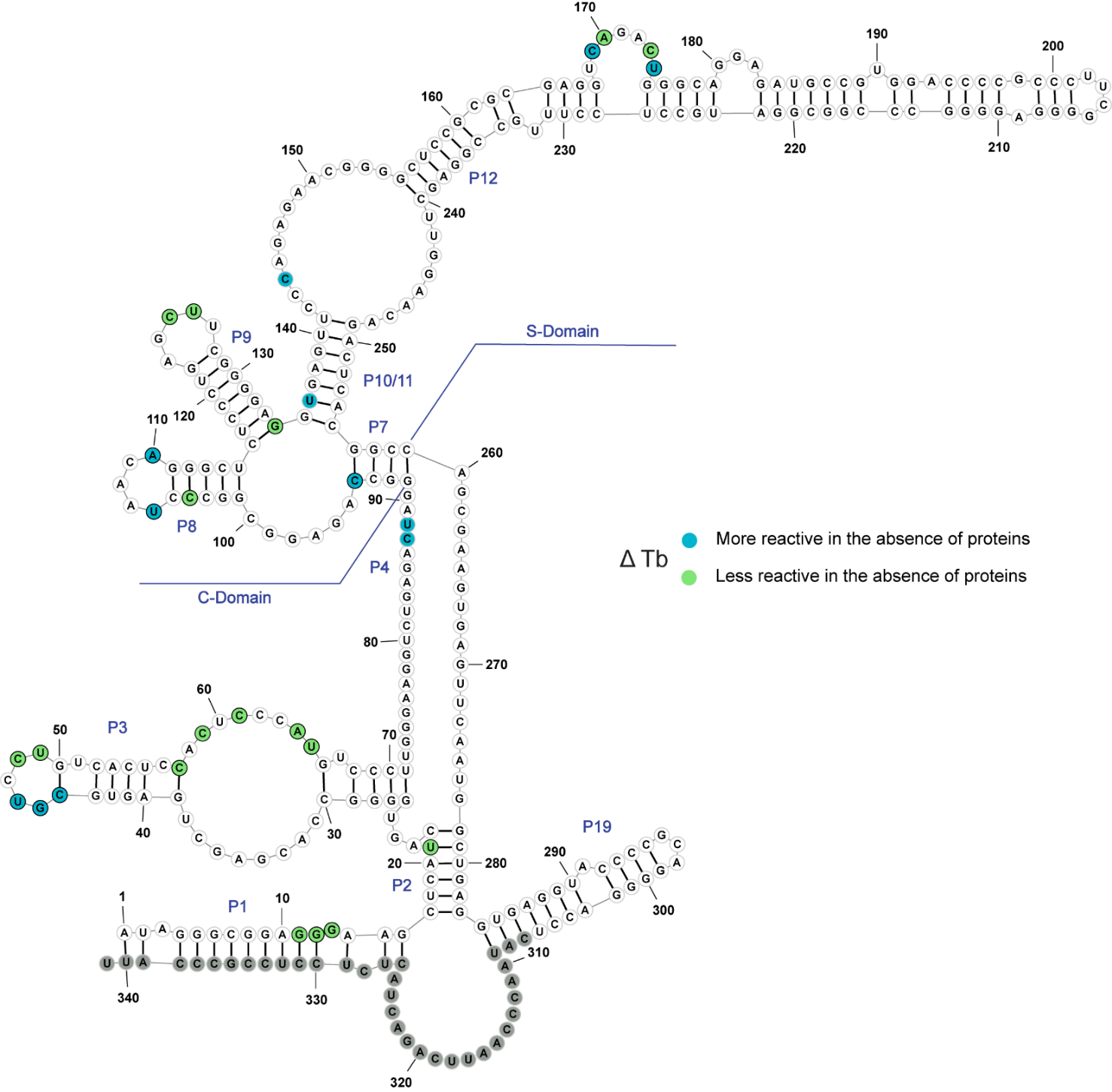


**Supplementary Figure 6.** Δ Tb analysis of human RNase P.

1. Secondary structure of RNase P displaying Δ Tb reactivities. For nucleotides in gray, no sequencing data is available. Source data are provided as a Source Data file.


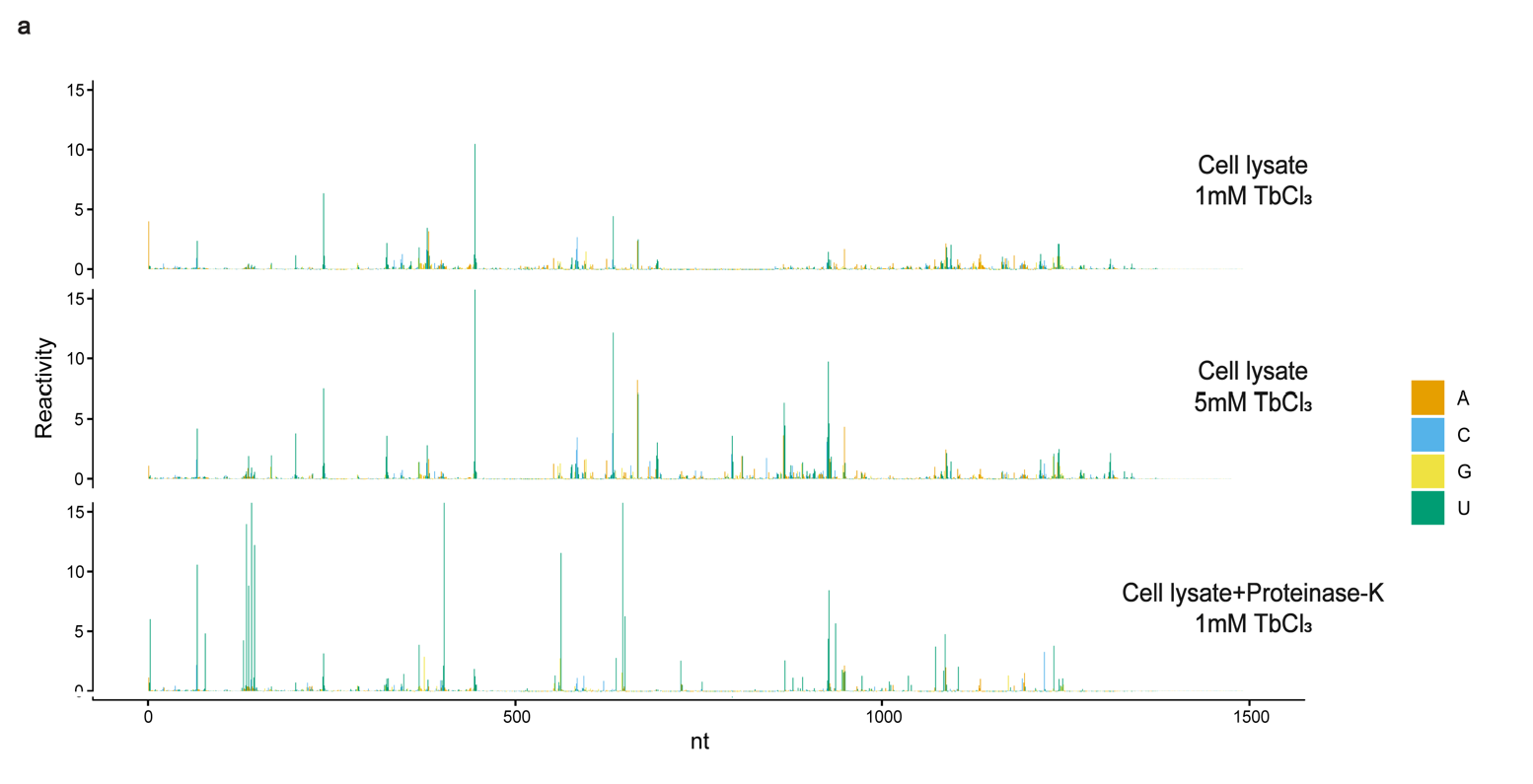


**Supplementary Figure 7.** Cell lysis probing of SARS-CoV-2 in the presence and absence of proteins.

1. Bar plot displaying reactivity values at the indicated concentrations and probing conditions. Source data are provided as a Source Data file.

**
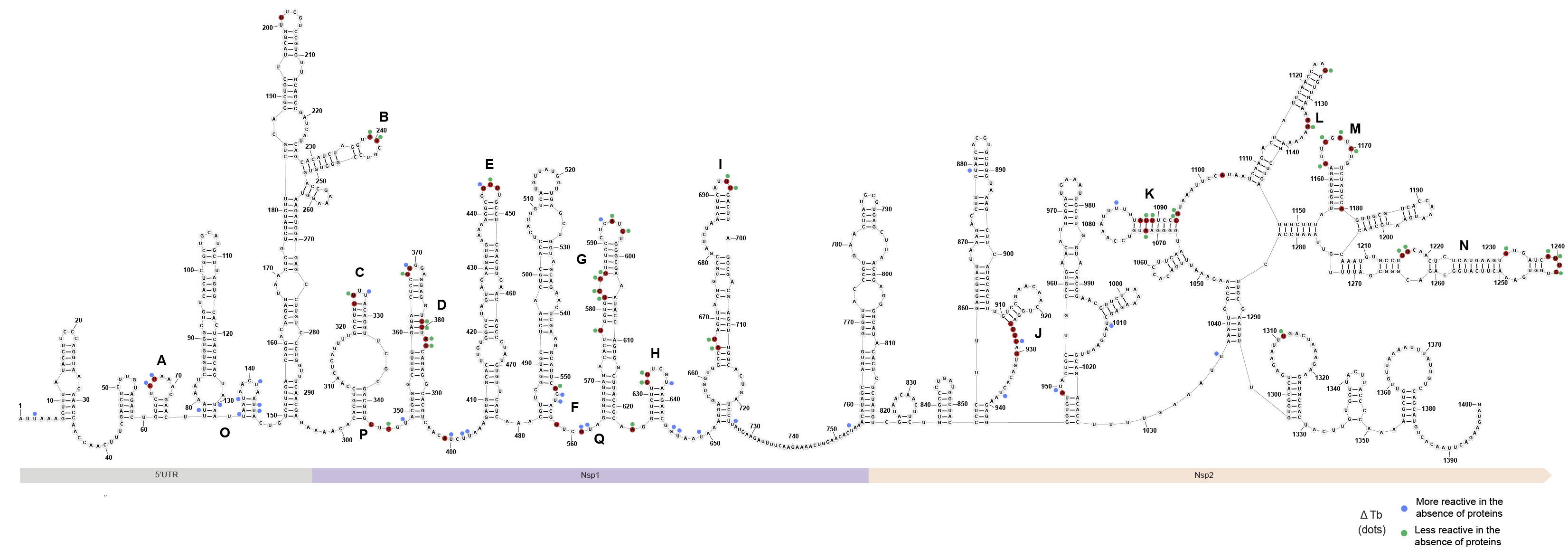
**

**Supplementary Figure 8.** Δ Tb analysis of 5’ end of SARS-CoV-2.

1. Secondary structure of the terminal 1400nt of SARS-CoV-2. Secondary structure of the SARS-CoV-2 displaying sites of strong Tb^3+^cleavage (red). Surrounding dots indicate Δ Tb reactivity and are grouped by regions A-Q. Source data are provided as a Source Data file.

**
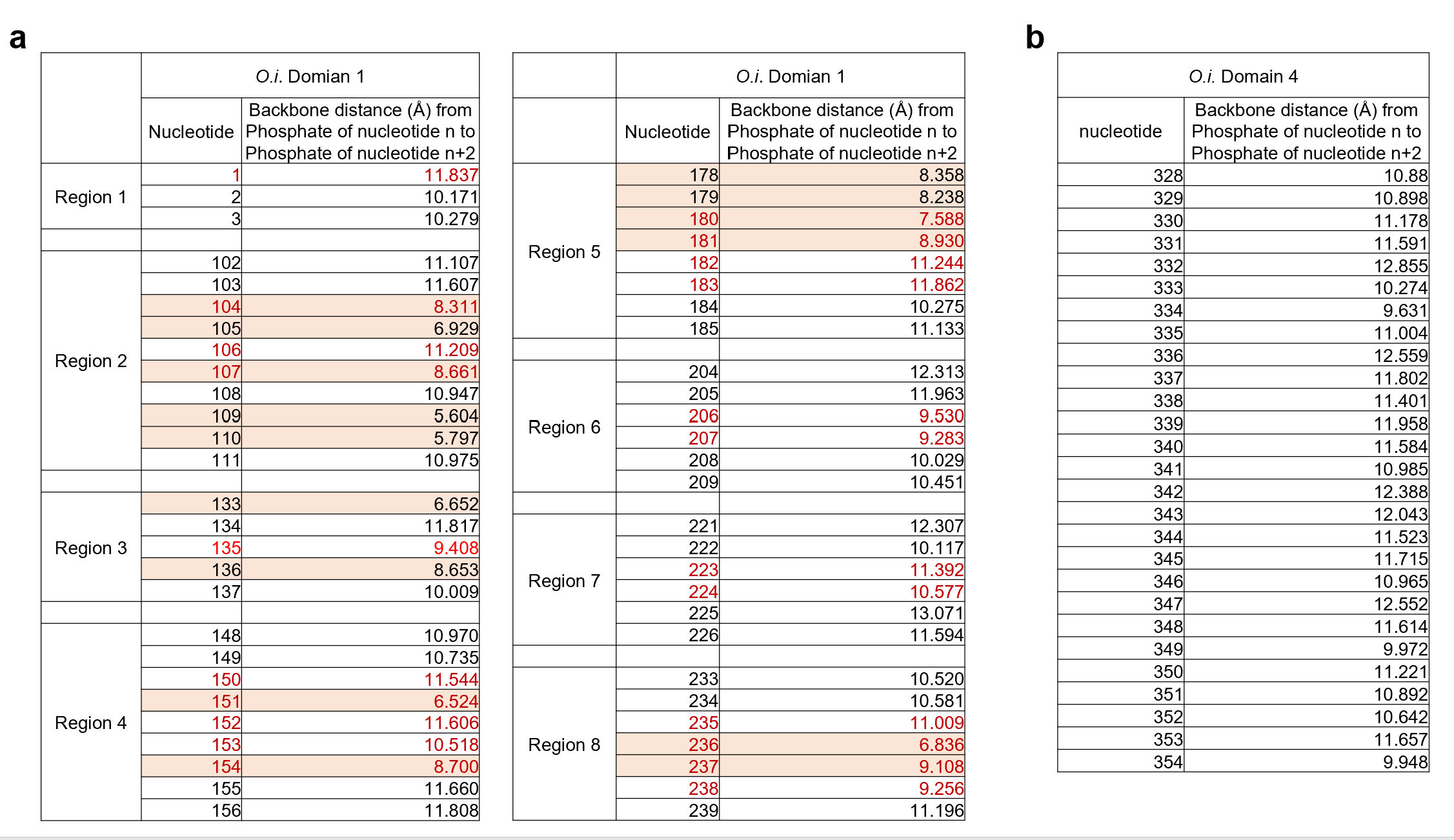
**


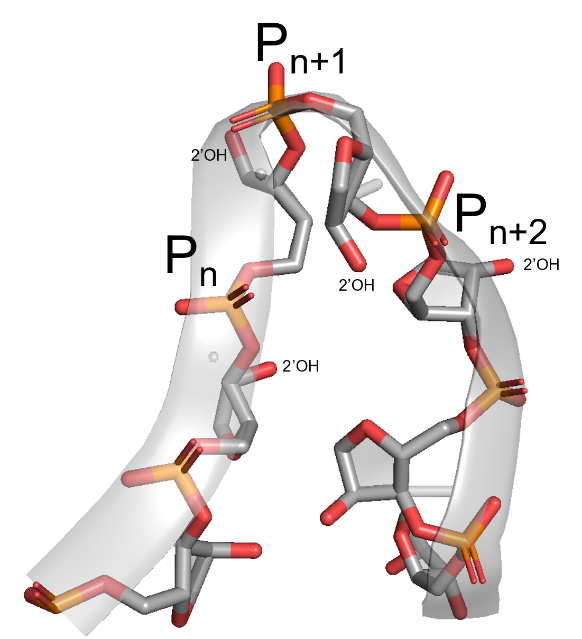
**c**

**Supplementary Figure 9.** Correlating Tb-seq signal with backbone phosphate distances for Group II *O.i.* intron.

1. Backbone distances (Å) for regions displaying Tb^3+^ cleavage. Distances were calculated from phosphate of nucleotide n to phosphate of nucleotide n+2 (P_n_ -> P_n+2_). In red text are sites which display strong Tb^3+^ cleavage. Highlighted boxes indicate distances deviating from values in a simple helix in *O.i.* domain 4^2^.
2. Backbone distances (Å) of helix in *O.i*. domain 4. Distances were calculated from phosphate of nucleotide n to phosphate of nucleotide n+2 (P_n_ -> P_n+2_). All values calculated from PDB [4E8M](https://www.rcsb.org/structure/4e8m).
3. Schematic displaying visual representation of sharpness metric.

Supplementary References

1 Sigel, R. K. O., Vaidya, A. & Pyle, A. M. Metal ion binding sites in a group II intron core. *Nat. Struct. Biol.* 7, 1111-1116 (2000).

2 Marcia, M. & Pyle, Anna M. Visualizing group II Intron catalysis through the stages of splicing. *Cell* 151, 497-507 (2012).
