# Supplementary material for: Systematic detection of tertiary structural modules in large RNAs and RNP interfaces by Tb-seq
Source: Nat Commun. 2023 Jun 9;14:3426. doi: 10.1038/s41467-023-38623-1 (PMC10255950; doi:10.1038/s41467-023-38623-1)
Supplement: Supplementary file 2 — Reporting Summary [file 41467_2023_38623_MOESM2_ESM.pdf]

nature portfolio

Corresponding author(s): Anna Marie Pyle

Last updated by author(s): May 7, 2023

Reporting Summary

Nature Portfolio wishes to improve the reproducibility of the work that we publish. This form provides structure for consistency and transparency in reporting. For further information on Nature Portfolio policies, see our Editorial Policies and the Editorial Policy Checklist.

Statistics

For all statistical analyses, confirm that the following items are present in the figure legend, table legend, main text, or Methods section.

n/a	Confirmed	
☐	☒	The exact sample size (*n*) for each experimental group/condition, given as a discrete number and unit of measurement
☐	☒	A statement on whether measurements were taken from distinct samples or whether the same sample was measured repeatedly
☒	☐	The statistical test(s) used AND whether they are one- or two-sided<br>*Only common tests should be described solely by name; describe more complex techniques in the Methods section.*
☒	☐	A description of all covariates tested
☒	☐	A description of any assumptions or corrections, such as tests of normality and adjustment for multiple comparisons
☒	☐	A full description of the statistical parameters including central tendency (e.g. means) or other basic estimates (e.g. regression coefficient) AND variation (e.g. standard deviation) or associated estimates of uncertainty (e.g. confidence intervals)
☒	☐	For null hypothesis testing, the test statistic (e.g. *F*, *t*, *r*) with confidence intervals, effect sizes, degrees of freedom and *P* value noted<br>*Give P values as exact values whenever suitable.*
☒	☐	For Bayesian analysis, information on the choice of priors and Markov chain Monte Carlo settings
☒	☐	For hierarchical and complex designs, identification of the appropriate level for tests and full reporting of outcomes
☒	☐	Estimates of effect sizes (e.g. Cohen's *d*, Pearson's *r*), indicating how they were calculated

Our web collection on statistics for biologists contains articles on many of the points above.

Software and code

Policy information about availability of computer code

Data collection	All sequencing data were acquired using a NextSeq 550 or NextSeq 2000 system.
Data analysis	All data were analyzed using the code in Sexton et al., 2017 and as described in the methods section of the manuscript. All secondary structures were visualized and drawn using Structure Editor, v1.0. All three-dimensional structure renderings were done using PyMOL Molecular Graphics System, v1.2r3pre, Schrödinger, LLC. Graphical displays were made using GraphPad Prism 8 or RStudio v1.2.5001. All gels were visualized using ImageJ 1.52a or ImageQuant TL v8.2.0.

For manuscripts utilizing custom algorithms or software that are central to the research but not yet described in published literature, software must be made available to editors and reviewers. We strongly encourage code deposition in a community repository (e.g. GitHub). See the Nature Portfolio guidelines for submitting code & software for further information.

Data

Policy information about availability of data

All manuscripts must include a data availability statement. This statement should provide the following information, where applicable:

- Accession codes, unique identifiers, or web links for publicly available datasets
- A description of any restrictions on data availability
- For clinical datasets or third party data, please ensure that the statement adheres to our policy

All FASTQ files generated in this study have been deposited in the SRA database under accession code PRJNA966800 [https://www.ncbi.nlm.nih.gov/sra/

PRJNA966800]. The processed data generated in this study are provided in the Supplementary Information/Source Data file. The accession codes for the structures used in this study are as follows: 4E8M[https://www.rcsb.org/structure/4e8m], 5A2Q[https://www.rcsb.org/structure/5A2Q], 6AHR [https://www.rcsb.org/structure/6AHR] .

Human research participants

Policy information about studies involving human research participants and Sex and Gender in Research.

Reporting on sex and gender	Not applicable
Population characteristics	Not applicable
Recruitment	Not applicable
Ethics oversight	Not applicable

Note that full information on the approval of the study protocol must also be provided in the manuscript.

Field-specific reporting

Please select the one below that is the best fit for your research. If you are not sure, read the appropriate sections before making your selection.

☒ Life sciences ☐ Behavioural & social sciences ☐ Ecological, evolutionary & environmental sciences

For a reference copy of the document with all sections, see nature.com/documents/nr-reporting-summary-flat.pdf

Life sciences study design

All studies must disclose on these points even when the disclosure is negative.

Sample size	Sample size of as n=2 was chosen for most experiments. A sample size in this case corresponds to biological replicates of full chemical probing data on intact RNAs.
Data exclusions	No data was excluded from our analyses.
Replication	Experiments were performed as independent biological replicates.
Randomization	Randomization was not relevant to a chemical probing study.
Blinding	Blinding was not relevant to to a chemical probing study.

Reporting for specific materials, systems and methods

We require information from authors about some types of materials, experimental systems and methods used in many studies. Here, indicate whether each material, system or method listed is relevant to your study. If you are not sure if a list item applies to your research, read the appropriate section before selecting a response.

Materials & experimental systems

n/a	Involved in the study	
☒	☐	Antibodies
☐	☒	Eukaryotic cell lines
☒	☐	Palaeontology and archaeology
☒	☐	Animals and other organisms
☒	☐	Clinical data
☒	☐	Dual use research of concern

Methods

n/a	Involved in the study	
☒	☐	ChIP-seq
☒	☐	Flow cytometry
☒	☐	MRI-based neuroimaging

Eukaryotic cell lines

Policy information about cell lines and Sex and Gender in Research

Cell line source(s)	Huh 7.5, Cells were a gift from Brett Lindenbach. Cell line was generated as described in Blight KJ, McKeating JA, Rice CM. Highly permissive cell lines for subgenomic and genomic hepatitis C virus RNA replication. J Virol. 2002 Dec;76(24):13001-14.

Authentication	Cells were generated and authenticated by Brett Lindenbach.
Mycoplasma contamination	Cell lines were not tested for Mycoplasma contamination
Commonly misidentified lines (See ICLAC register)	No commonly misidentified cell lines were used in the study.
